# Mapping Malaria Vector Habitats in West Africa: Drone Imagery and Deep Learning Analysis for Targeted Vector Surveillance

**DOI:** 10.3390/rs15112775

**Published:** 2023-05-26

**Authors:** Fedra Trujillano, Gabriel Jimenez Garay, Hugo Alatrista-Salas, Isabel Byrne, Miguel Nunez-del-Prado, Kallista Chan, Edgar Manrique, Emilia Johnson, Nombre Apollinaire, Pierre Kouame Kouakou, Welbeck A. Oumbouke, Alfred B. Tiono, Moussa W. Guelbeogo, Jo Lines, Gabriel Carrasco-Escobar, Kimberly Fornace

**Affiliations:** 1 Health Innovation Laboratory, Institute of Tropical Medicine “Alexander von Humboldt”, Universidad Peruana Cayetano Heredia, Lima 15102, Peru; 2 School of Biodiversity, One Health & Veterinary Medicine, University of Glasgow, Glasgow G12 8QQ, UK; 3 Department of Engineering and Computer Science, Faculty of Science and Engineering, Sorbonne University, 75005 Paris, France; 4 Escuela de Posgrado Newman, Tacna 23001, Peru; 5 Science and Engineering School, Pontificia Universidad Católica del Perú (PUCP), Lima 15088, Peru; 6 Department of Infection Biology, London School of Hygiene & Tropical Medicine, London WC1E 7HT, UK; 7 Peru Research, Development and Innovation Center (Peru IDI), Lima 15076, Peru; 8 The World Bank, Washington, DC 20433, USA; 9 Centre on Climate Change and Planetary Health, London School of Hygiene & Tropical Medicine, London WC1E 7HT, UK; 10 Centre National de Recherche et de Formation sur le Paludisme, Ouagadougou 01 BP 2208, Burkina Faso; 11 Institute Pierre Richet, Bouake 01 BP 1500, Côte d’Ivoire; 12 Innovative Vector Control Consortium, Liverpool School of Tropical Medicine, London L3 5QA, UK; 13 Scripps Institution of Oceanography, University of California San Diego, La Jolla, CA 92093, USA; 14 Saw Swee Hock School of Public Health, National University of Singapore and National University Health System, Singapore 119077, Singapore

**Keywords:** malaria vector, deep learning, image classification, drone images, epidemiological control

## Abstract

Disease control programs are needed to identify the breeding sites of mosquitoes, which transmit malaria and other diseases, in order to target interventions and identify environmental risk factors. The increasing availability of very-high-resolution drone data provides new opportunities to find and characterize these vector breeding sites. Within this study, drone images from two malaria-endemic regions in Burkina Faso and Côte d’Ivoire were assembled and labeled using open-source tools. We developed and applied a workflow using region-of-interest-based and deep learning methods to identify land cover types associated with vector breeding sites from very-high-resolution natural color imagery. Analysis methods were assessed using cross-validation and achieved maximum Dice coefficients of 0.68 and 0.75 for vegetated and non-vegetated water bodies, respectively. This classifier consistently identified the presence of other land cover types associated with the breeding sites, obtaining Dice coefficients of 0.88 for tillage and crops, 0.87 for buildings and 0.71 for roads. This study establishes a framework for developing deep learning approaches to identify vector breeding sites and highlights the need to evaluate how results will be used by control programs.

## 1 Introduction

Land use changes, such as agricultural expansion, can create new aquatic habitats suitable for breeding sites for mosquito vectors, which transmit malaria and other diseases [1]. The identification of such water bodies can be vital to disease control programs, allowing vector control teams to perform targeted malaria control by larval source management (LSM) [2]. LSM targets the immature aquatic stages of disease vectors through environmental, chemical or biological modification of the larval habitat, with the overall aim of reducing adult mosquito populations [3]. While traditional approaches have relied on ground-based surveys, Earth Observation (EO) data, such as drone and satellite data, are increasingly utilized to identify potential breeding sites rapidly and target control measures [2–6]. EO data additionally provide new opportunities to characterize mosquito habitats and monitor changes in these habitats in response to environmental changes [7].

Obtaining actionable information from EO data requires classifying imagery into relevant habitat types. The specific classes of interest are highly dependent on the local vector ecology. For example, *An. gambiae* breed in small or transient, often man-made, water bodies across a wide range of habitat types, including puddles on roads, agricultural irrigation such as rice paddies, sunlit rivers and streams and quarries or construction sites [8–10]. As these breeding sites are often temporary and may be difficult to directly observe (e.g., under trees or in small water bodies), identifying important land types where breeding sites occur can be an important proxy to target vector control measures. Further information on the locations of houses and other buildings may also aid planning for vector control campaigns. Additionally, the type of information required when classifying EO data depends on the end use. While a vector control program may benefit from knowing the probability of a large area containing habitat types, ecological and epidemiological studies aiming to identify risk factors for vector breeding sites may require more detailed segmentation approaches to characterize the shape and configuration of different habitat types, so as to identify where these breeding sites are likely to occur [11–17].

In contrast to *An. gambiae*, *An. funestus* typically breeds in large semipermanent or permanent water bodies, often characterized by emergent vegetation [2,18–21]. Despite *An. funestus* being Africa’s second most important malaria vector, and although the size and permanence of the species’ breeding sites should make them intuitively easy to locate, *An. funestus* breeding sites are notoriously difficult to detect [21]. When compared to the small and transient *An. gambiae* breeding sites, the large and stable characteristics of the *An. funestus* breeding sites make them a suitable target for identification using EO techniques. These water bodies have often been associated with agricultural practices, including rice cultivation, irrigation canals and ditches, pastures and cultivated swamps [22–27]. A review of the available published literature on *An. funestus* breeding ecology [2] identified key characteristics of *An. funestus* breeding sites, which include irrigated and non-irrigated forms of agriculture and savannah landscapes. They also identified land classes that are likely to exist in landscapes where humans and malaria vectors overlap, but which are not necessarily associated with the breeding cycle, such as roads, buildings and other features of the built environment. Additional information on infrastructure, including the locations of houses and other buildings, can aid planning interventions for vector control campaigns.

For these applications, it is critical that EO data are collected simultaneously as ground-based vector surveys or are recent enough to provide actionable information for control programs. *Anopheles’* breeding sites are difficult to detect from coarse, freely available, satellite-based EO data such as Sentinel or LandSat, with aquatic habitats often small (<1 m), vegetated or obscured depending on local vector ecology. Additionally, breeding sites may be temporary or exist in landscapes that are rapidly modified, requiring temporally accurate EO data to link with ground-based surveys. This can be challenging with satellite-based EO sources where data are collected infrequently (weekly or monthly) and limited by cloud cover or other factors [28,29]. This has led to the increased use of EO data with high spatial and temporal resolutions, such as user-defined imagery collected by drones (unmanned aerial vehicles or UAVs) or daily commercial satellite data (e.g., Planet). These data typically have a low spectral resolution, limiting the utility of traditional pixel-based approaches requiring data measured outside the visible spectrum [30,31]. Alternatively, deep learning approaches, such as convolutional neural networks (CNNs), have revolutionized image analysis by efficiently analyzing image textures, patterns and spectral characteristics using self-learning artificial intelligence approaches to identify features in complex environments [16,32–34].

Multiple approaches have been applied in identifying habitats and their characteristics from EO imagery for operational use by vector-borne disease control programs. In Malawi, Stanton et al. [3] assessed approaches to identifying the aquatic habitats of larval-stage malaria mosquitoes. They assessed geographical object-based image analysis (GeoOBIA), which groups contiguous pixels into *objects* based on prespecified pixel characteristics. The objects were classified by a random-forest-supervised classification and demonstrated strong agreement with test samples, successfully identifying larval habitat characteristics with a median accuracy of 98%. Liu et al. [35] developed a framework for mapping the spatial distribution of suitable aquatic habitats for the snail hosts of the debilitating parasitic disease Schistosomiasis along the Senegalese River Basin. A deep learning U-Net model was built to analyze high-resolution satellite imagery and to produce segmentation maps of aquatic vegetation. The model produced predictions of snail habitats with higher accuracy than commonly used pixel-based classification methods such as random forest. Hardy et al. [36] developed a novel approach to classify and extract malaria vector larval habitats from drone imagery in Zanzibar, Tanzania. This used computer vision to assist manual digitization. This approach significantly outperformed supervised classification approaches, which were unsuitable for mapping potential vector larval habitats in the study region based on accuracy scores. Examples of methods for other applications, data sources and the performance of different classification techniques are summarized in Table 1.

Building on these methods, we aimed to develop and validate deep learning approaches to identify land classes associated with the breeding sites of malaria mosquito vectors in West Africa. Data were assembled from multiple sites in Burkina Faso and Côte d’Ivoire to develop an approach able to generalize to different malaria-endemic landscapes. We developed a land classification system based on habitats of interest for the breeding ecology of the malaria vector An. funestus and An. gambiae, which are present in the study sites. We used RGB drone images from the study sites to build a training dataset and to implement two CNN-based frameworks using the U-Net and attention U-Net architectures to identify features of interest for *Anopheles* breeding. The specific objectives of this study were to (i) collect and assemble a labeled dataset of drone images in malaria-endemic areas in Côte d’Ivoire and Burkina Faso; (ii) develop a protocol to label land classes of interest based on the local vector ecology; (iii) assemble a labeled dataset for each class and (iv) train, validate and test the U-Net and attention U-Net deep learning architectures. The final algorithms were assessed based on the performance in predicting the presence of the classes of interest in test images using the best model after cross-validation.

## 2 Materials and Methods

### 2.1 Drone Mapping

**Figure 1:**
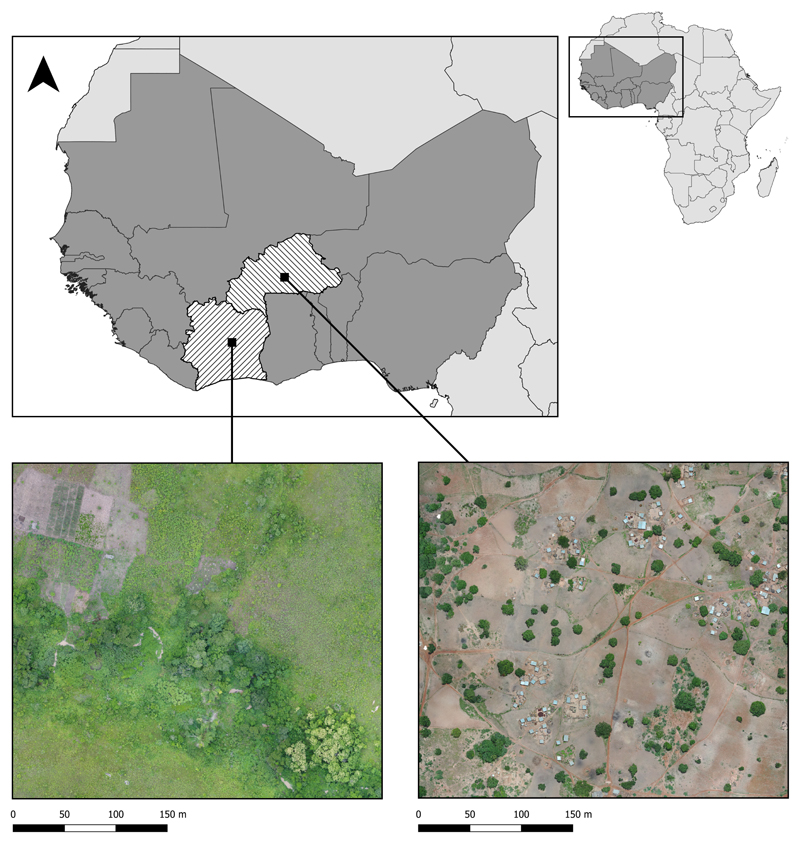
Drone image collection sites with example drone imagery from each site.

Drone surveys were conducted in two malaria-endemic sites in West Africa—Saponé, Burkina Faso and Bouaké, Côte d’Ivoire—where the incidence of malaria (per 1000 population at risk) is 389.9 and 287, respectively (the World Bank: https://data.worldbank.org/indicator/SH.MLR.INCD.P3(accessed on 19 April 2023)). Both sites are rural, with extensive small-scale agriculture and highly seasonal rainfall and malaria transmission patterns. Saponé is located 45 km south-west of Ouagadougou, Burkina Faso and has reported an extremely high malaria prevalence of predominantly *Plasmodium falciparum* [42]. The main malaria vector in this site is *An. gambiae* s.l., with low densities of other species also reported. Between November 2018 and November 2019, with an average temporal resolution of 5 months, fixed-wing (Sensefly eBee) and quadcopter (DJI Phantom 4 Pro) drones were used to collect 26 RGB images at 2–10 cm per pixel resolution. Similarly, in Bouaké, we conducted targeted drone surveys from June to August 2021 using a DJI Phantom 4 Pro drone to collect RGB data at a 2 cm per pixel resolution, as described by [2]; 77 images were used in this study. This area was also rural and dominated by small-scale agriculture; however, this site had different vector compositions, including high densities of *An. funestus*. For both sites, drone images were processed using Agisoft Metashape Professional (Agisoft: https://www.agisoft.com/(accessed on 19 April 2023)). The steps performed were photo alignment (using high accuracy, selecting the generic preselection, reference preselection and adaptive camera model fitting options; the key point was set to 40,000), building a dense point cloud (high quality and moderate depth filtering), building a digital elevation model (extrapolated option) and finally performing the orthomosaic generation. The drone images covered a 11.52 km^2^ and 30.42 km^2^ area for Burkina Faso and Côte d’Ivoire, as illustrated in Figure 1. Both sites were dominated by agriculture, mainly yams, cassava, cashews, peanuts and maize.

### 2.2 Image Labeling and Development of Labeled Dataset

We identified specific land cover classes of interest associated with *Anopheles* breeding sites, including water bodies and irrigated agricultural land types [2]. Within these settings, crops, roads and tillage areas were identified as land types with a high probability of containing small water bodies where *An. gambiae* breeds, the predominant vector in the Burkina Faso site. For *An. funestus*, a dominant vector in areas of the Cote d’Ivoire site, we differentiated between vegetated and non-vegetated water bodies as *An. funestus* is most commonly identified only in vegetated water bodies. We additionally identified habitat types associated with human activities, including roads and buildings. The final land cover classes of interest for this analysis included vegetated and non-vegetated water bodies, irrigated crops (planted vegetation with no tree cover), tillage (land cleared for planting crops), buildings and roads. While this classification cannot specifically identify whether or not an area is an *Anopheles* breeding site, this provides the basis for targeting future entomological surveys and wider epidemiological studies on how landscape impacts malaria transmission.

**Figure 2:**
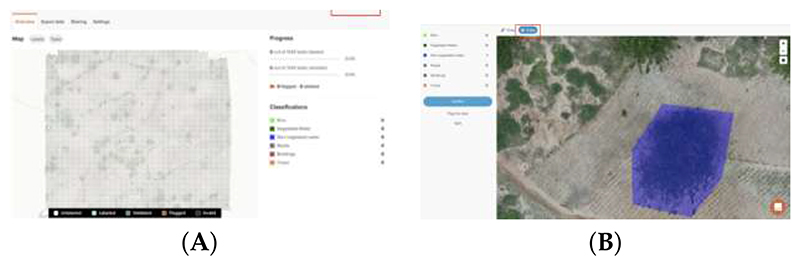
Example of image labeling using GroundWork. (**A**) Grid over an image for labeling. (**B**) Polygon selection.

**Figure 3:**
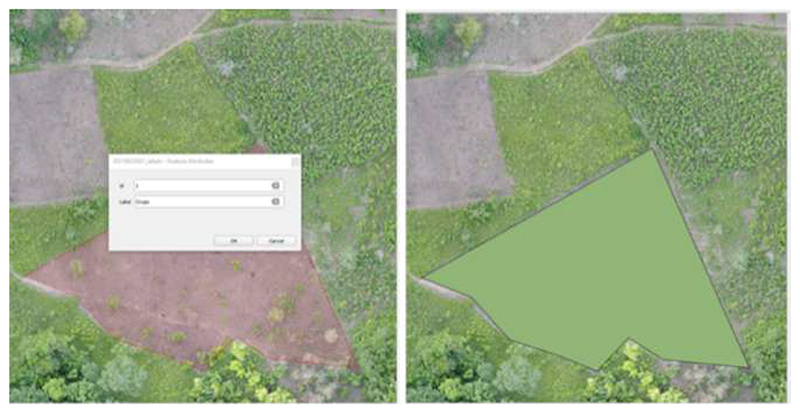
Example of image labeling using QGIS.

To identify these classes using a supervised deep learning approach, we first needed to assemble a dataset of harmonized labeled images. We manually labeled a total of 103 drone acquisitions to generate gold-standard (i.e., labeled images) masks for each of the specific land classes. Trained personnel familiar with the study area validated the labels. This process was performed using two different tools: GroundWork (GroundWork: https://groundwork.azavea.com (accessed on 19 April 2023)) and QGIS (QGIS: https://qgis.org/site/forusers/download.html(accessed on 19 April 2023)). The former is a cloud-based licensed tool where the images are uploaded for labeling. In this cloud-based interface, a grid is overlaid in the image as illustrated in Figure 2A. The user is then able to select one of the predefined classes to assign the corresponding class over the images, as shown in Figure 2B. While this tool has a streamlined workflow to facilitate labeling, this required internet access, had a limited data allowance and was not suitable in all contexts. Therefore, we also used QGIS, which, on the other hand, is a Geographic Information System (GIS) open-source desktop application that allows one to input geo-referenced images and annotate them manually using polygons (Figure 3). Images labeled with each software were randomly selected. The obtained polygons from both tools were checked to detect invalid polygons and were corrected manually.

**Figure 4:**
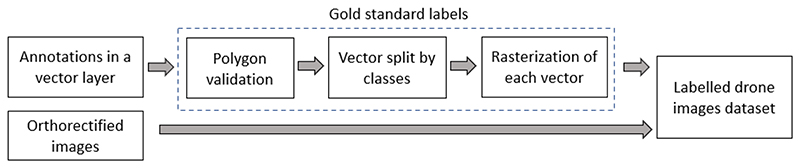
Gold-standard (ground truth) mask process.

**Figure 5:**
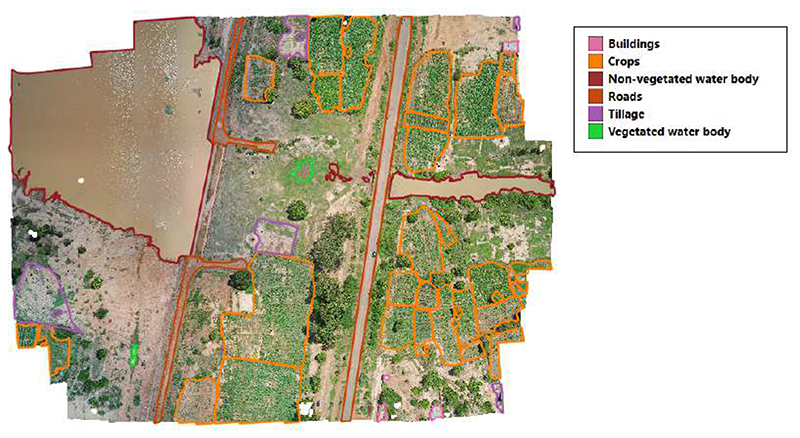
Image example from Burkina Faso.

Once all the images were labeled, ground truth masks were created for the supervised image classification. As shown in Figure 4, first, we create a subset of vector layers, one for each class. Then, we rasterized the vector layers to create a separate raster image for each class. The land class presence depended on the acquisition site’s characteristics. For example, Figure 5 shows a labeled region from Burkina Faso where all the land classes are present; however, this was not the case for all the images. Images that did not contain labeled polygons were not considered in this study.

We built six land class datasets: crops, tillage, roads, buildings, vegetated water bodies and non-vegetated water bodies (defined at the beginning of this section). For each dataset, we selected only the drone images that contained labels of the corresponding land class. From each dataset, we randomly selected images for training, validation and testing. To avoid bias related to the training data, we considered using a 3-fold cross-validation scheme.

**Figure 6:**
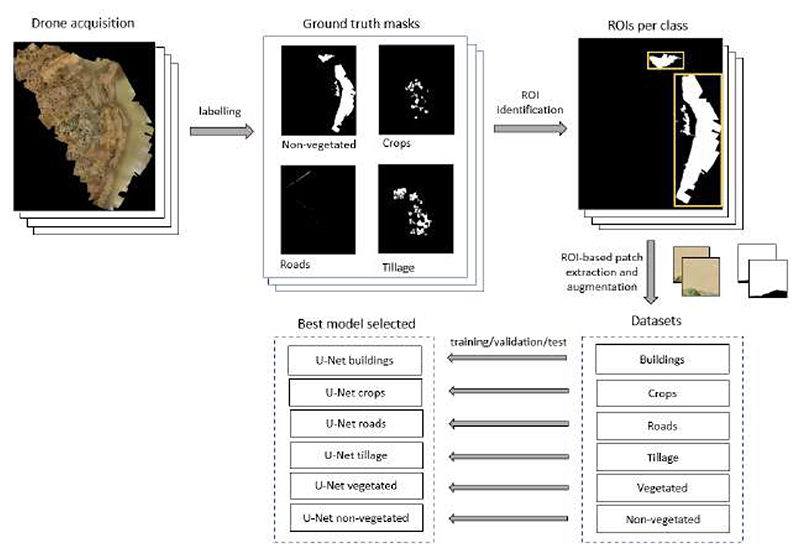
Classification methodology schema.

Due to GPU memory constraints, the entire drone image and its corresponding labels were split into several patches, which were used to train, validate and test the deep learning models. In addition, because of the variable extension of each labeled polygon, splitting the image in a grid pattern resulted in a highly unbalanced dataset where the background predominated over the class of interest. Instead, we used a new approach for data patching and augmentation based on ROI shifting, described in [43], to avoid this imbalance. This method also prevented potential bias caused by the network focusing on the background instead of the class of each patch. The augmentation aimed to train the models robustly in the presence of variable neighborhood context information. Thus, we identified the ROIs and framed them in rectangles, which could contain one or more polygons, as illustrated in Figure 6. Patches of 256 × 256 and 512 × 512 pixels were extracted from these rectangles and assigned to each training, validation and testing dataset. A summary of the number of images and patches in each dataset is reported in Table 2. For each patch size, we report in Table 3 the average percentage of the class present in the dataset. This was computed, per patch, as the ratio of the pixels belonging to each class and the background pixels. Patches with classes representing less than 10% (256 × 256) or 20% (512 × 512) of the patch size were eliminated from the datasets. The total number of patches obtained for each class differed by study site; while Burkina Faso contained more tillage areas, Côte d’Ivoire had more irrigated crops. Considering both sites, water body datasets contained the least number of patches. On average, non-vegetated water bodies, tillage and crops covered greater percentages of patches as these classes were more likely to be larger.

### 2.3 Algorithm Development

We developed a multi-step approach to classifying multiple land classes from patches, as shown in Figure 6. Following the dataset preparation, two deep learning segmentation models were selected: U-Net and attention U-Net. U-Net is a widely used architecture for semantic segmentation tasks. This method relies on the upsampling technique, which increases an image’s dimensions (i.e., the number of rows and/or columns). Thus, the present method builds on a conventional network with successive layers by using up-sampling operators to replace pooling operations, which implies using contraction and expansion paths (i.e., encoder and decoder, respectively). The contraction part reduces the spatial dimensions in every layer and increases the channels. Meanwhile, the expansive part increases the spatial dimensions while reducing the channels. Finally, using also skip connections between the encoder and decoder, the spatial dimensions are restored to predict each pixel in the input image. An important modification in U-Net is that many feature bands are in the upper sampling part, allowing the network to propagate context information to higher-resolution layers. Consequently, the expanding trajectory is more or less symmetric to the contracted part and produces a U-shaped architecture [44]. Attention U-Net adds a self-attention gating module in every skip connection of the U-Net architecture, without increasing the computation overhead. These modules are incorporated to improve the sensitivity and accuracy and add visual explainability to the network. The improvement is performed by focusing on the features of the regions of interest rather than the background [45,46].

Regarding the computational features used in this study, data preparation and deep learning experiments were executed on an 8-core Intel(R) Xeon E5-2686 @ 2.3 GHz CPU with 60 GiB RAM and one 16 GiB RAM Nvidia Tesla V100 GPU on the Amazon Elastic Compute Cloud service (AWS).

### 2.4 Evaluation Metrics

To quantitatively assess the similarities between the predicted and gold-standard object areas, we used the Dice coefficient. This divides two times the area of overlap by the total number of pixels in both images, as shown in Equation (1).

(1) $$DICE=\frac{2\times \left|A\cap B\right|}{\left|A\cap B\right|+\left|A\cup B\right|}$$

The Dice coefficient takes values from 0 to 1, in which 1 represents a complete match between the ground truth and the prediction. We additionally calculated the precision and recall metrics, which are computed based on true positives (TP), false positives (FP), true negatives (TN) and false negatives (FN), as described in Equations (2) and (3), respectively.

(2) $$\text{Precision}=\frac{\text{TP}}{\text{TP}+\text{FP}}$$

(3) $$\text{Recall}=\frac{\text{TP}}{\text{TP}+\text{FN}}$$

Based on the aforementioned elements, we performed different experiments, which are described in the following section.

## 3 Results

As the number of ROIs in every drone image was not the same, the number of patches (generated from the ROIs) in each fold varied from class to class. As a result, we used up to 90% of the CPU RAM capacity in the experiments containing the higher number of patches. This computational load was due to caching the data and annotations in CPU RAM prior to moving the batches to the GPU RAM in the training, validation and test phases. This approach reduces the number of CPU–GPU data transfers, which can intensively impact the training time. The average training and cross-validation time was approximately 12 h.

The U-Net and attention U-Net architectures were used to classify the different classes organized in sets of patches of 256 × 256 and 512 × 512 pixels in size using a three-fold cross-validation procedure. Table 4 shows the results for the U-Net using patches with a size of 256 × 256 pixels. For vegetated water bodies, one of our primary classes of interest, the model reached its highest Dice score at 0.68 in the first fold and an average of 0.63. Non-vegetated water bodies had a higher Dice score of 0.75 and 0.58 on average, showing the worst performance among all the classes. Crops, tillage and buildings had the best overall performance, above 0.80 in all validation sets, followed by roads, which reached 0.71. The same U-Net architecture was also trained with patches of 512 × 512 pixels in size. For both training approaches, all classes achieved comparable results; however, the model trained with 256 × 256 pixel size patches outperformed, on average, the 512 × 512 model in every class. The detailed table showing the performance of the 512 × 512 pixel size model is provided in the Supplementary Information.

**Figure 7:**
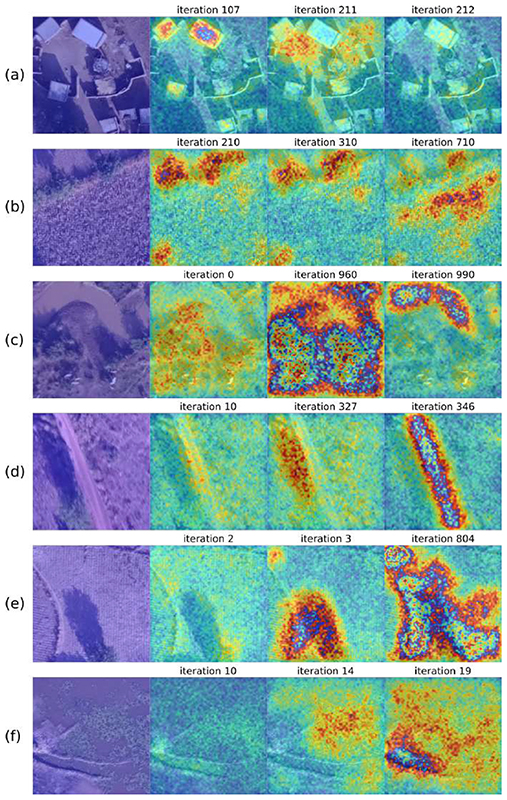
Evolution of the training process using the attention U-Net architecture for patches of size 256 × 256 pixels. (**a**) Buildings. (**b**) Crops. (**c**) Non-vegetated water bodies. (**d**) Roads. (**e**) Tillage. (**f**) Vegetated water bodies.

Similarly to the U-Net experiments, we trained the attention U-Net with patches of 256 × 256 pixels. The evolution of the training using a heatmap in a jet color scale is shown in Figure 7. The areas where the network found relevant features for segmentation are displayed in red, while the blue areas correspond to less essential regions. The first column shows the original patch, and the following columns are the heatmaps according to an iteration number indicated above; however, they correspond to different epochs. In general, for non-vegetated water body (Figure 7c), road (Figure 7d) and vegetated water body (Figure 7f) patches, we noticed that, as the number of iterations increased, the network focused more on the areas of interest for learning. Nevertheless, in the case of buildings (Figure 7a), the initial iteration focused more on the construction than the final one because it corresponded to a different epoch and batch, meaning that iteration 212 corresponded to an early epoch where the network was not fully updated or a batch with a different data distribution than the patch analyzed. Another important aspect to highlight is that, in some iterations, the network concentrated not on the class but on the shadows of the patch, such as in Figure 7d iteration 327 and Figure 7d iteration 3.

The quantitative results to evaluate the performance of the attention U-Net using patches of 256 × 256 pixels are reported in Table 5. Comparing these results with the ones obtained with the 256 × 256 U-Net model, we observe that the vegetated and non-vegetated water body classes had improved performance. Despite this, the first fold of the non-vegetated water body class showed a low Dice value (below 5%), meaning that the training was unstable across the three folds and may have impacted the inference process due to the different data distributions present in the datasets. Therefore, cross-validation provided insights into the robustness and stability of the trained models.

We also trained the attention U-Net using patches of 512 × 512 pixels. However, we used a subset of patches per class ranging from 5% to 20% to test the model performance on the vegetated and non-vegetated water body, crop and building classes. Although it showed an improvement in the water body classes compared to the 256 × 256 pixel U-Net model using the best fold as a reference, the standard deviation calculated after the cross-validation was higher, meaning that the network was not entirely stable. For instance, in the non-vegetated class, the Dice score ranged from 0.1 to 0.91 in the attention U-Net 512 × 512 pixel model. We report all results for this last experiment in the Supplementary Information.

**Figure 8:**
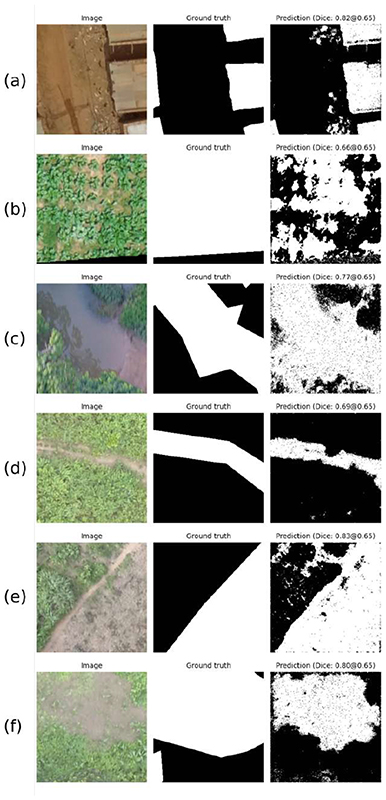
Predictions using the U-Net architecture for patches of size 256 × 256 pixels. (**a**) Buildings. (**b**) Crops. (**c**) Non-vegetated water bodies. (**d**) Roads. (**e**) Tillage. (**f**) Vegetated water bodies.

We selected the U-Net 256 × 256 pixel model to evaluate the predicted mask as it was the most stable and robust network. Figure 8 shows the inference results for one sample patch (taken from the test dataset) per class. The first column shows the original test patch. The second column shows the gold standard in white and the background in black, whereas the third column is the network prediction, where each pixel is depicted in white if it belongs to the corresponding class with a probability higher than 0.65. The patch’s Dice score is reported above each predicted mask. Figure 8a shows the buildings accurately distinguished over the soil region. In contrast, qualitative results for the crop class in Figure 8b show that the network predicts as crops more regions of soil between the leaves rather than the actual crop. This may be explained by the imprecise annotations (i.e., mask almost covering 100% of the patch area) seen in the gold standard. Figure 8c presents a water body’s segmentation despite the shadows and blurriness of the patch. Figure 8a,d show a road and a vegetated water body, respectively. In both cases, the network outperformed the manual annotation qualitatively. Finally, the tillage model output shown in Figure 8e predicted not only the corresponding class but also areas of soil that were not prepared for cultivation.

## 4 Discussion

This study highlights the utility of deep learning approaches to identify potential mosquito habitats using high-resolution RGB imagery. We developed a workflow and methodology to assemble and process labeled training data to implement deep learning algorithms to automatically detect malaria vectors’ potential habitats. Although the performance, as measured by the Dice coefficient, was low for some classes, the classifier did consistently detect the presence of specific classes within drone imagery. In fact, we identified that the relevant information for the end-user needs, in this case, is to identify the presence of a particular type of land cover rather than the boundary delimitation of the class. Overall, this work establishes a framework to apply artificial intelligence tools to support vector-borne disease control.

Our proposed methodology builds on a growing body of literature using deep learning approaches and remote sensing data to identify priority areas in implementing disease control measures. Compared to other studies using deep learning algorithms with multispectral satellite imagery to detect vector habitats, our study had lower predictive power [35], most likely due to the limited information in RGB images. In addition, the annotation process (i.e., manual labeling) is a factor that we need to consider. For example, Figure 8f shows an example of the human labeling error in not encompassing the water bodies’ boundaries. However, despite these errors on the training labels, the network performs better in segmenting the pixels that belong to this class. The qualitative differences in the manual and predicted labels result in a lower Dice score. Rather than relying solely on manual annotations, which can be imprecise, unsupervised learning approaches or region-growing approaches may result in more accurate ground truths and higher-performing models [36].

As shown in Figure 8f, our proposal using the U-Net architecture achieved better qualitative results when segmenting certain classes. In order to understand deeply where the network was focusing its attention when dealing with this task, we used the attention U-Net architecture. The results provided a clear view of which pixels were being used in the segmentation process. This introduced a level of visual interpretability of the training process and allowed us to propose a methodology to leverage the attention maps to refine manual annotations.

**Figure 9:**
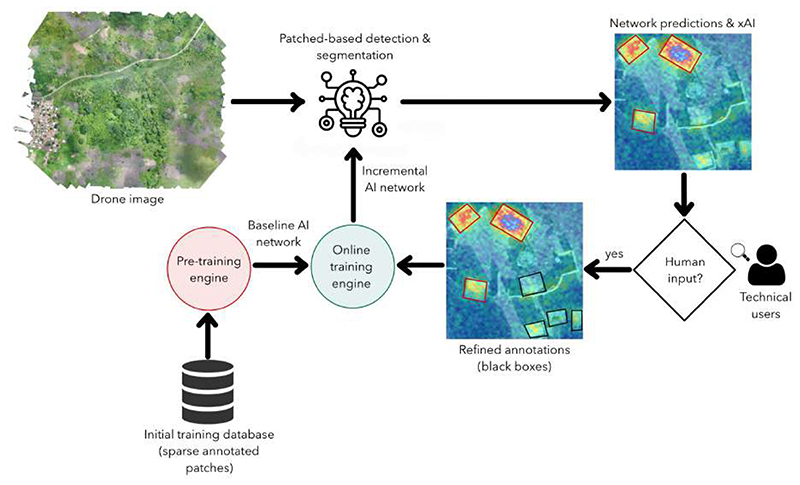
Future proposal: human-supervised tool for improved drone labeling. The idea is adapted from [43,47].

This tool can be improved in the future by including human supervision, as proposed in Figure 9. Initially, we need a set of manually annotated patches extracted from several drone images used in the pre-training engine. The model obtained after this process could be used to segment and detect structures in a new drone image. The network will output its predictions as attention maps (heatmaps with pixel probabilities). A user will then evaluate the predictions and determine if there are missing objects or if the boundaries of the detected objects are correctly segmented. These new annotations will then be used as inputs to an online training engine, improving the knowledge of the original deep learning model. This procedure should reduce the imprecision of manual annotations and allow the model to learn incrementally from new samples introduced by different users. In addition, the feedback loop should also help when there are similar qualitative characteristics (imaging features) in different class patches—a challenging process regarding data cleaning procedures.

We could also observe a difference in performance using different patch sizes. Ideally, larger patches should allow the network to extract information from the object’s surroundings and identify the borders of the objects detected, such as the buildings. However, this additional information may need to be clarified in some cases. A deeper analysis, including multi-resolution deep learning models, could provide a better understanding of the features needed for better segmentation of the classes proposed in this study.

Deep learning approaches based on open-access satellite data can provide a more efficient and cost-effective means for vector control programs to identify priority areas for field surveys and targeted interventions. Larval source management is an important component in the toolkit for controlling mosquito-borne diseases, particularly in endemic contexts with persistent insecticide resistance [36]; however, identifying aquatic breeding sites is both time- and resource-intensive and can be biased by reliance on prior knowledge, convenience or assumptions. Based on the Dice score obtained, we found that the presence of specific habitat classes could be consistently detected within drone imagery, including vegetated and non-vegetated water bodies, tillage, crops and roads. By delineating certain areas within a large, gridded landscape with a high probability of containing potential vector breeding habitats, deep learning algorithms could facilitate the more targeted planning and implementation of larvicidal activities. For example, vector control programs can use this to focus finite resources on narrower areas for entomological field-based surveys or anticipate the scale of larvicide requirements for a given area. More broadly, knowledge of roads and building locations can be used to plan interventions. For example, clustered buildings in close proximity to breeding sites are important targets for indoor residual spraying against adult malaria vectors. Importantly, this approach is generalizable and could be used in a range of vector-borne disease-endemic contexts to identify the presence of habitats of interest that are relevant to the local land cover and local vector ecology.

One of the key advantages of drone data is that they allow user-defined time points to characterize features over time. This study was predominantly cross-sectional, aiming to classify land types from labeled drone images from specific points in time. As one of the key aims was to categorize potential breeding sites for the malaria vector *An. funestus*, which breeds in large, semipermanent to permanent water bodies, breeding sites are less likely to vary throughout the seasons. However, these methods could be repeated to reclassify potential habitats for specific seasons or time points. This could be particularly informative in mapping seasonal changes in breeding site availability or monitoring agricultural activities that expand different habitat types.

Additionally, this study highlights the importance of identifying how the classified information will be used. While we assessed model performance using the Dice coefficient, these metrics describe pixel-level classification accuracy. In some cases, this may be appropriate, such as when an epidemiological study needs to identify the precise outline of a water body. However, in many cases, these scores do not reflect the utility of the classifier. For example, a control program may only need to know where a potential breeding site is located and the relative size in order to plan larvicidal activities.

This study has several important limitations. While we used data from multiple sites in West Africa, these do not indicate the full range of habitats within this region or seasonal changes. Future studies could integrate data from other sources to develop more representative datasets. Additionally, limited amounts of ground-truthed data were available from these study sites, and there were insufficient data on larvae presence or absence to predict whether specific land classes contained *Anopheles* larvae. If data were available, this framework could be extended to predict the presence or absence of specific species.

Despite these limitations, this study developed a methodology to automatically detect potential mosquito breeding sites. Although data labeling is highly labor-intensive, this classifier can rapidly analyze RGB drone images collected using small, low-cost drones. Similarly, as deep learning methods are self-learning, additional datasets will likely improve the performance and applicability of these methods. Future work could develop more user-friendly interfaces to support the uptake of these methods. Altogether, this study sets out a useful framework to apply deep learning approaches to RGB drone imagery.

## 5 Conclusions

This study proposed a methodology to automatically spotlight high-resolution RGB drone images of the West Africa land cover to detect malaria vectors’ potential habitats. After manual image annotation, images were cut into patches of size 256 × 256 and 512 × 512 pixels. Later, U-Net-based and attention U-Net-based algorithms were applied to automatically identify buildings, roads, water bodies, crops and tillage. Finally, the best model was selected from the different experiments performed based on the Dice score. Although we obtained promising results in identifying buildings, roads and water bodies, crops and tillage still represent a challenge, which will be explored in future work. Nevertheless, we have demonstrated that our proposal is pertinent in helping experts to create tools to avoid the proliferation of mosquito breeding sites.

## Supplementary Material

Supplementary Material

## Figures and Tables

**Table 1:** Summary of land cover classification methods.

Location	Application	Imaging Source	Method	Result
Senegal River, West Africa [35]	Mapping snails’ aquatic habitats	Satellite: 8-band World View 2 for training UAV: used to assess labeling	Semantic segmentation using U-Net 8-band + GLCM features	Accuracy 4 classes Test: 82.7% 4 classes hold-out validation: 96.5%
Anbandegi, Korea [37]	Crop classification Kimchi cabbage	UAV: green, red, NIR bands	SVM and RF. Using GLCM features to reduce noise	Overall accuracy 4 classes: 98.72%
Queensland, Australia [38]	Ground coverage Wheat crops	UAV: RGB Real image set (RISs) Synthetic image set (SISs)	Two-step approach: per-pixel segmentation, sub-pixel segmentation using regression tree classifier	RMSE RISs:<6% SISs: <5%
Pardubice, Czech Republic [39]	Land cover identification near small water body	UAV: RGB	Comparing 8 different vegetation indexes	Visual comparison Best performance: NGRDI, GLI2, VARI
Chengdu, China [40]	Mapping vegetation, impervious surface and soil in urban environment	Satellite: Landsat-8 Operational Land Imager (OLI)	Applied multiple criteria spectral mixture analysis (MCSMA) with multi-step approach for spectral unmixing	RMSE Vegetation: 0.143 Soil: 0.170 Impervious: 0.151
Ghana and South Sudan [41]	Semantic segmentation of crops in Africa	Satellite: Sentinel-1 (VV and VH), Sentinel-2 (10 bands) and Planet Scope (RGB + NIR)	Compared: 2D U-Net + CLSTM and 3D CNN using multi-temporal images	Accuracy South Sudan: 2D U-Net 88.7%, 3D 90% Ghana: 2D U-Net 65.7%, 3D 63.5%

**Table 2:** Number of images and patches by class for training, validation and test.

Class	# Drone Images	# Train/Val Images	# Train/Val Patches		# Test Images	# Test Patches	
			256 × 256	512 × 512		256 × 256	512 × 512
Buildings	48	36	7441	1324	12	4835	714
Crops	93	69	229,522	58,738	24	64,357	16,440
Roads	60	45	38,799	3908	15	14,330	2106
Tillage	42	31	86,988	22,817	11	39,025	10,264
Non-vegetated	37	27	9194	2232	10	79	1
Vegetated	20	15	4660	1107	5	564	125

**Table 3:** Summary of the percentage of the class per patch in each category.

Burkina Faso								
Patch size	256 × 256				512 × 512			
Class	patches	min (%)	mean (%)	max (%)	patches	min (%)	mean (%)	max (%)
Buildings	6584	10.01	33.72	100	778	20.05	35.81	87.9
Crops	14,440	10.00	76.70	100	3662	20.00	71.23	100.0
Roads	42,810	10.00	29.51	100	4513	20.00	36.72	100.0
Tillage	121,952	10.00	75.49	100	32,089	20.00	70.35	100.0
Non-vegetated	7734	10.09	91.73	100	1900	20.08	89.51	100.0
Vegetated	251	10.17	65.89	100	58	20.49	61.03	100.0
Côte d’Ivoire								
Patch size	256 × 256				512 × 512			
Class	patches	min (%)	mean (%)	patches	min (%)	mean (%)	max (%)
Buildings	5692	10.0	46.5	100	1260	20.0	35.6	82
Crops	279,439	10.0	79.5	100	71,516	20.0	74.2	100
Roads	10,319	10.0	31.4	100	1501	20.0	26.7	96
Tillage	4061	10.0	69.4	100	992	20.1	62.3	100
Non-vegetated	1539	10.1	55.3	100	333	20.0	56.3	100
Vegetated	5646	10.0	62.3	100	1107	20.1	58.5	100

**Table 4:** Results of the classification process using a U-Net architecture for 256 × 256 pixels patch size, where the best fold is reported in bold font. The results are reported in terms of cross-validation (CV), false positives (FP), false negatives (FN), true negatives (TN), true positives (TP), precision, recall and Dice.

Class	CV	FP	FN	TN	TP	Precision	Recall	Dice
Vegetated water body	1	0.17	0.15	0.15	0.53	0.75	0.78	**0.68**
	2	0.25	0.19	0.20	0.37	0.59	0.66	0.56
	3	0.21	0.12	0.22	0.45	0.68	0.78	0.65
	Avg.	0.21	0.15	0.19	0.45	0.67	0.74	0.63
Tillage	1	0.10	0.03	0.13	0.74	0.88	0.96	**0.88**
	2	0.10	0.09	0.15	0.66	0.87	0.88	0.82
	3	0.11	0.05	0.13	0.71	0.87	0.93	0.86
	Avg.	0.10	0.06	0.14	0.70	0.87	0.92	0.85
Roads	1	0.09	0.07	0.63	0.21	0.70	0.74	0.70
	2	0.06	0.06	0.71	0.17	0.73	0.75	**0.71**
	3	0.16	0.15	0.51	0.17	0.52	0.53	0.43
	Avg.	0.10	0.09	0.62	0.18	0.65	0.67	0.61
Non-vegetated water body	1	0.02	0.54	0.06	0.38	0.95	0.41	0.50
	2	0.21	0.26	0.26	0.27	0.57	0.51	0.48
	3	0.22	0.03	0.18	0.57	0.72	0.96	**0.75**
	Avg.	0.15	0.28	0.17	0.41	0.74	0.63	0.58
Crops	1	0.10	0.06	0.10	0.74	0.87	0.93	0.86
	2	0.13	0.04	0.09	0.75	0.85	0.95	0.86
	3	0.09	0.04	0.10	0.76	0.89	0.95	**0.88**
	Avg.	0.11	0.05	0.10	0.75	0.87	0.94	0.86
Building	1	0.04	0.07	0.51	0.38	0.89	0.84	0.81
	2	0.04	0.08	0.57	0.31	0.88	0.80	0.76
	3	0.04	0.03	0.54	0.38	0.90	0.92	**0.87**
	Avg.	0.04	0.06	0.54	0.35	0.89	0.85	0.81

**Table 5:** Results of the classification process using an attention U-Net architecture used for 256 × 256 pixels patch size, where the best fold is reported in bold font. The results are reported in terms of cross-validation (CV), false positives (FP), false negatives (FN), true negatives (TN), true positives (TP), precision, recall, F1-score and Dice.

Class	CV	FP	FN	TN	TP	Precision	Recall	Dice
Vegetated water body	1	0.27	0.08	0.15	0.49	0.64	0.85	0.67
	2	0.28	0.11	0.17	0.44	0.61	0.81	0.64
	3	0.08	0.14	0.24	0.53	0.81	0.72	**0.70**
	Avg.	0.21	0.11	0.19	0.49	0.69	0.79	0.67
Tillage	1	0.06	0.25	0.18	0.51	0.85	0.66	0.67
	2	0.11	0.23	0.13	0.53	0.80	0.68	**0.69**
	3	0.03	0.55	0.27	0.15	0.77	0.20	0.27
	Avg.	0.07	0.34	0.19	0.40	0.81	0.51	0.54
Roads	1	0.15	0.12	0.53	0.20	0.70	0.67	0.58
	2	0.15	0.10	0.48	0.27	0.71	0.69	**0.60**
	3	0.35	0.06	0.40	0.19	0.51	0.77	0.46
	Avg.	0.22	0.09	0.47	0.22	0.64	0.71	0.55
Non-vegetated water body	1	0.10	0.58	0.30	0.02	0.36	0.03	0.04
	2	0.06	0.10	0.02	0.82	0.91	0.88	**0.85**
	3	0.20	0.05	0.26	0.49	0.71	0.90	0.72
	Avg.	0.12	0.24	0.19	0.44	0.66	0.60	0.54
Crops	1	0.07	0.17	0.14	0.62	0.85	0.76	0.75
	2	0.04	0.27	0.13	0.55	0.89	0.66	0.72
	3	0.16	0.04	0.05	0.75	0.81	0.94	**0.84**
	Avg.	0.09	0.16	0.11	0.64	0.85	0.79	0.77
Building	1	0.03	0.06	0.56	0.36	0.91	0.84	**0.85**
	2	0.03	0.07	0.53	0.37	0.91	0.82	0.83
	3	0.21	0.06	0.41	0.32	0.65	0.83	0.66
	Avg.	0.09	0.06	0.50	0.35	0.82	0.83	0.78

## Data Availability

For reproducibility purposes, the python code is available at https://github.com/healthinnovation/aerial-image-analysis (accessed on 19 April 2023).

## Abbreviations

EO: Earth observation
UAV: unmanned aerial vehicle
CNN: convolutional neural network
DP: deep learning
TAD: technology-assisted digitizing
GLCM: gray-level co-occurrence matrix
RGB: red, green and blue
RMSE: root mean square error
MCSMA: multiple-criteria spectral mixture analysis
RNN: recurrent neural network
ReLU: rectified linear
CPU: central processing unit
RAM: random access memory
