# Supplementary material for: Mapping Malaria Vector Habitats in West Africa: Drone Imagery and Deep Learning Analysis for Targeted Vector Surveillance
Source: Remote Sens (Basel). Author manuscript; Available in PMC 2023 Jun 15. (PMC7614662; doi:10.3390/rs15112775)
Supplement: Supplementary Material [file EMS176389-supplement-Supplementary_Material.pdf]

Supplementary Materials: Deep Learning Image Analysis Tools to Identify Malaria Vector Habitats in West Africa

Fedra Trujillano [1,8], Gabriel Jimenez Garay[1], Hugo Alatrista-Salas[2,3]*, Isabel Byrne[4], Miguel Nunez-del-Prado[3,5], Kallista Chan[4,7], Edgar Manrique[1], Emilia Johnson[8], Nombre Apollinaire[9], Pierre Kouame Kouakou[10], Achille Welbeck Oumbouke[4,11], Alfred Tiono[7], Moussa Guelbeogo[7], Jo Lines[4,7], Gabriel Carrasco-Escobar [1,12], Kimberly Fornace[7,8,13]

Class	CV	FP	FN	TN	FP	Precision	Recall	Dice
Vegetated water body	1	0.31	0.17	0.19	0.33	0.52	0.66	0.54
	2	0.16	0.17	0.20	0.47	0.73	0.73	0.65
	3	0.31	0.06	0.16	0.47	0.60	0.89	**0.67**
	Avg.	0.26	0.13	0.18	0.42	0.61	0.76	0.62
Tillage	1	0.11	0.04	0.18	0.67	0.86	0.94	**0.88**
	2	0.09	0.11	0.20	0.59	0.86	0.84	0.81
	3	0.11	0.06	0.18	0.65	0.85	0.92	0.86
	Avg.	0.10	0.07	0.19	0.64	0.86	0.90	0.85
Roads	1	0.07	0.10	0.63	0.20	0.74	0.65	**0.66**
	2	0.06	0.09	0.69	0.16	0.76	0.65	0.66
	3	0.20	0.17	0.44	0.19	0.50	0.53	0.46
	Avg.	0.11	0.12	0.59	0.18	0.67	0.60	0.59
Non-vegetated water body	1	0.01	0.75	0.09	0.15	0.94	0.16	0.22
	2	0.36	0.01	0.01	0.62	0.64	0.98	**0.74**
	3	0.44	0.00	0.01	0.55	0.56	0.99	0.68
	Avg.	0.27	0.26	0.03	0.44	0.71	0.63	0.55
Crops	1	0.09	0.09	0.17	0.65	0.87	0.88	0.84
	2	0.12	0.04	0.15	0.68	0.85	0.94	0.87
	3	0.07	0.07	0.17	0.69	0.90	0.91	**0.88**
	Avg.	0.10	0.07	0.17	0.67	0.87	0.91	0.86
Building	1	0.04	0.07	0.61	0.29	0.88	0.80	**0.82**
	2	0.05	0.08	0.60	0.27	0.85	0.77	0.75
	3	0.08	0.05	0.58	0.29	0.80	0.86	0.81
	Avg.	0.06	0.07	0.60	0.28	0.84	0.81	0.79

Table S1: Results of the classification process using the U-Net architecture used for 512x512 pixels patch size, where the best fold is reported in bold font. The results are reported in terms of cross validation (CV), false positives (FP), false negatives(FN), true negatives (TN), true positives (TP), precision, recall, and Dice.

Class	CV	FP	FN	TN	TP	Precision	Recall	Dice
Vegetated water body	1	0.44	0.01	0.03	0.52	0.54	0.98	0.66
	2	0.20	0.07	0.31	0.43	0.69	0.84	**0.73**
	3	0.07	0.16	0.29	0.48	0.81	0.67	0.66
	Avg.	0.24	0.08	0.21	0.48	0.68	0.83	0.68
Non-vegetated water body	1	0.08	0.51	0.37	0.05	0.36	0.08	0.11
	2	0.07	0.06	0.03	0.84	0.92	0.93	**0.91**
	3	0.08	0.49	0.30	0.13	0.71	0.21	0.28
	Avg.	0.08	0.35	0.23	0.34	0.66	0.41	0.43
Crops	1	0.05	0.26	0.24	0.45	0.77	0.58	0.59
	2	0.05	0.26	0.24	0.44	0.77	0.58	**0.59**
	3	0.03	0.54	0.31	0.12	0.62	0.16	0.18
	Avg.	0.04	0.35	0.26	0.34	0.72	0.44	0.45
Building	1	0.02	0.05	0.64	0.29	0.93	0.84	**0.86**
	2	0.03	0.06	0.62	0.30	0.91	0.83	0.85
	3	0.04	0.06	0.61	0.30	0.86	0.83	0.81
	Avg.	0.03	0.06	0.62	0.30	0.90	0.83	0.84

Table S2: Results of the classification process using the Attention U-Net architecture used for 512x512 pixels patch size, where the best fold is reported in bold font. The results are reported in terms of cross validation (CV), false positives (FP), false negatives(FN), true negatives (TN), true positives (TP), precision, recall, and Dice.

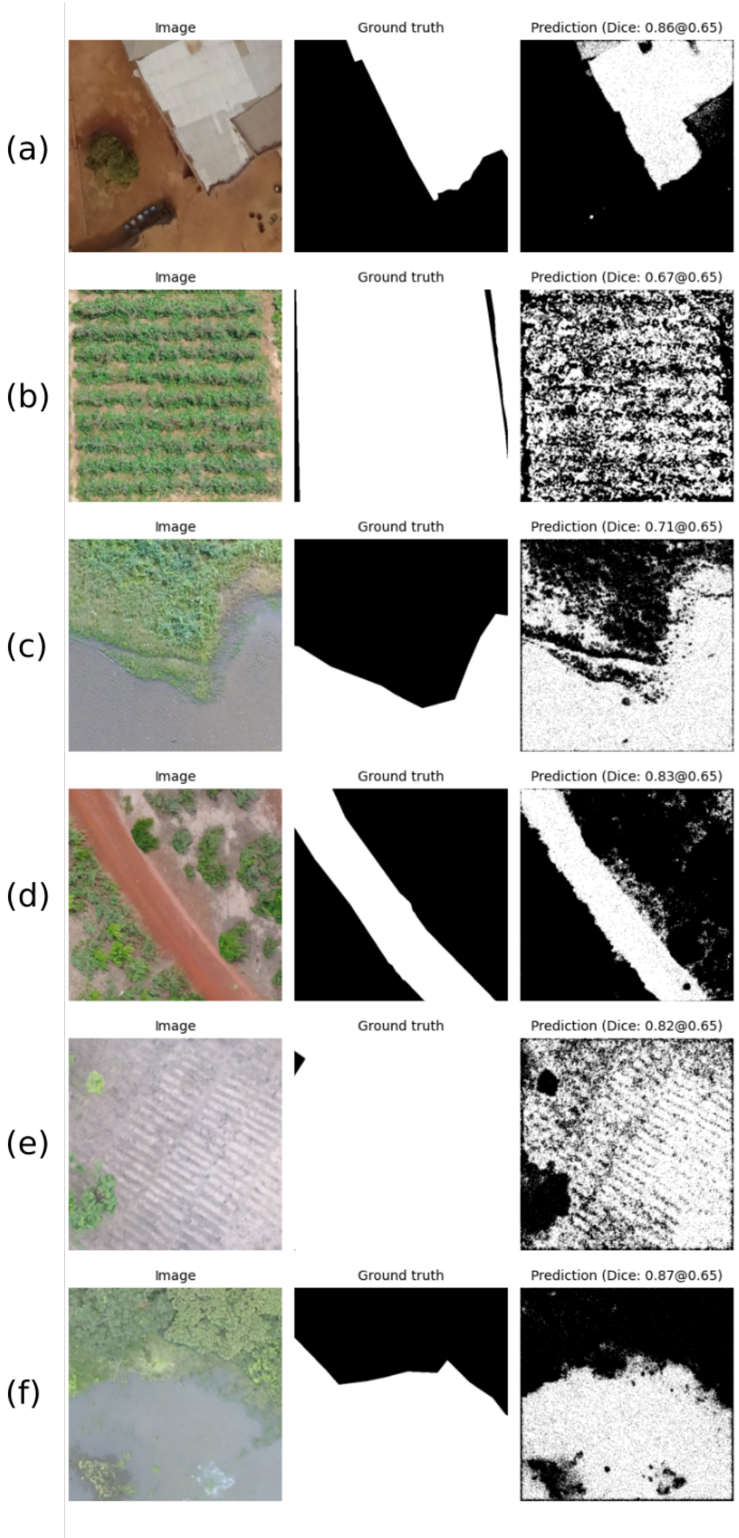

Figure S1. Predictions using the UNet architecture for patches of size 512×512 pixels. (a) Buildings. (b) Crops. (c) Non-vegetated water bodies. (d) Roads. (e) Tillage. (f) Vegetated water bodies.

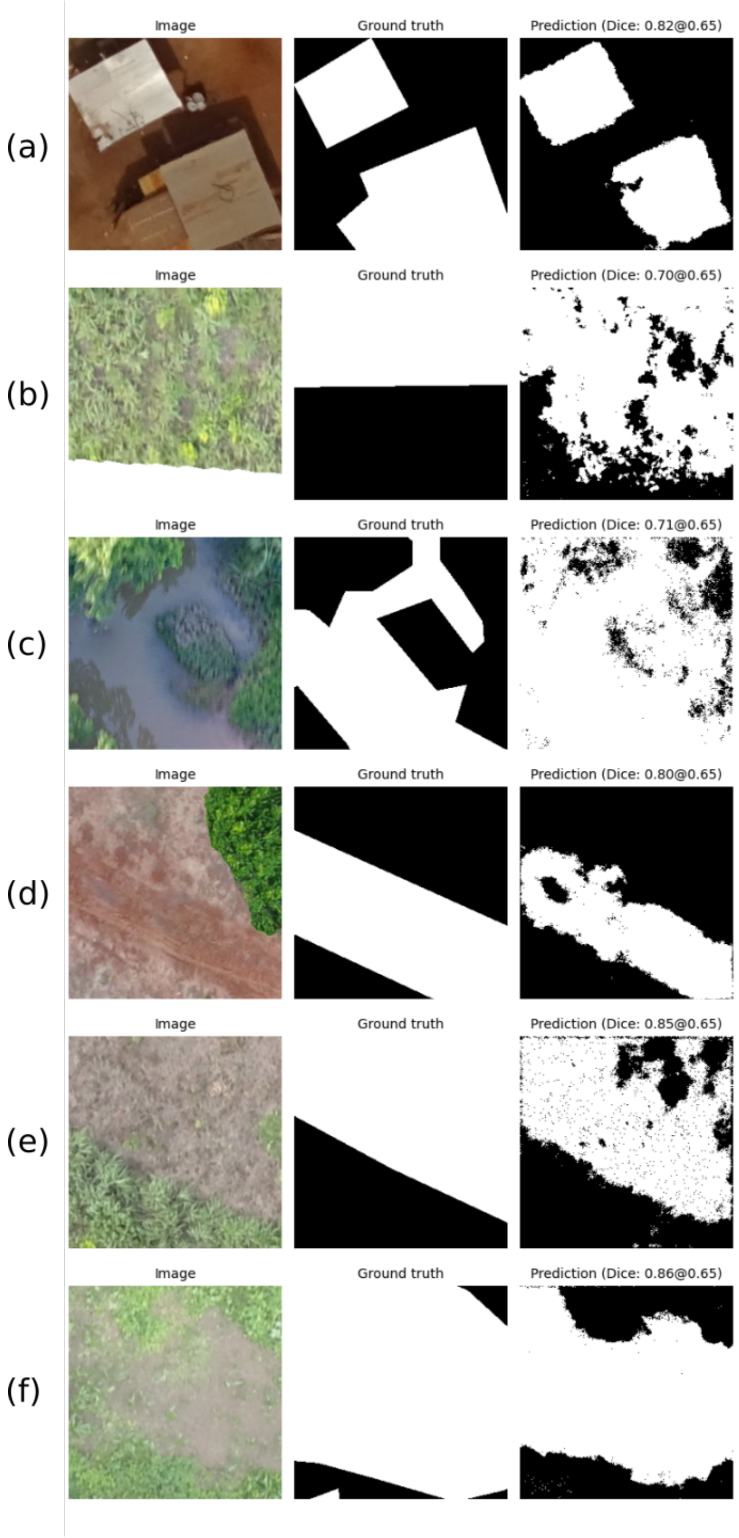

Figure S2. Predictions using the Attention U-Net architecture for patches of size 256×256 pixels. (a) Buildings. (b) Crops. (c) Non-vegetated water bodies. (d) Roads. (e) Tillage. (f) Vegetated water bodies.

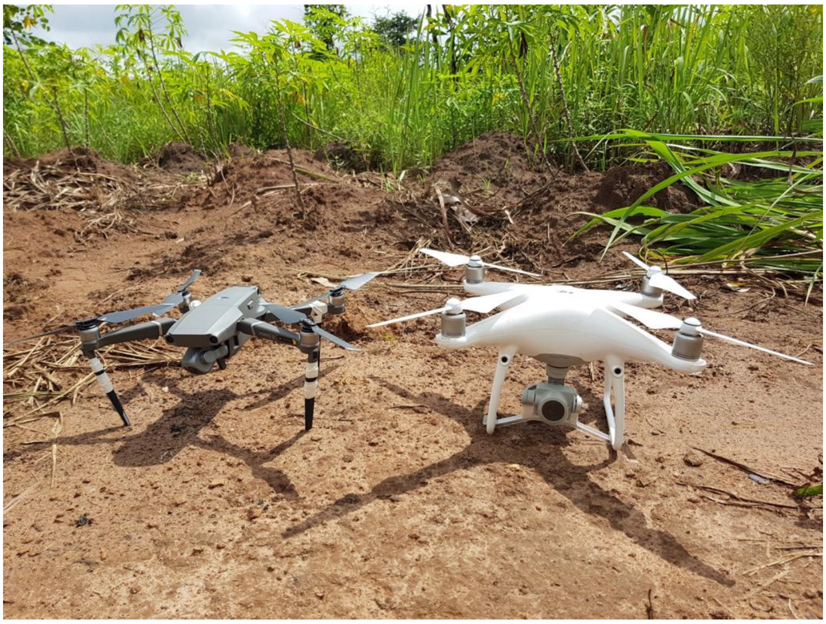

Figure S3. Drone mapping in Cote d'Ivoire
